# Efficiency of Multiple Extraction Solvents on Antioxidant, Cytotoxic, and Phytotoxic Potential of *Taraxacum officinale* (L.) Weber ex F.H. Wigg. from Poonch Valley, Azad Kashmir, Pakistan

**DOI:** 10.1155/2022/5118553

**Published:** 2022-05-24

**Authors:** Wasim Akhtar, Ghazanfar Ali, Nadia Ashraf, Iram Fatima, Waqas Khan Kayani, Hamayun Shaheen, Mohammed M. Ghoneim, Mohamed A. Abdelgawad, Ahmed Khames

**Affiliations:** ^1^Department of Botany, University of Azad Jammu and Kashmir Muzaffarabad, Muzaffarabad, Pakistan; ^2^Department of Biotechnology, University of Azad Jammu and Kashmir Muzaffarabad, Muzaffarabad, Pakistan; ^3^Department of Biotechnology, Fatima Jinnah Women University Rawalpindi, Rawalpindi, Pakistan; ^4^Department of Biotechnology, Faculty of Sciences, University of Kotli, Kotli, Azad Jammu and Kashmir, Pakistan; ^5^Department of Pharmacy Practice, Faculty of Pharmacy, AlMaarefa University, Ad Diriyah 13713, Saudi Arabia; ^6^Pharmacognosy and Medicinal Plants Department, Faculty of Pharmacy (Boys), Al-Azhar University, Cairo 11884, Egypt; ^7^Department of Pharmaceutical Chemistry, College of Pharmacy, Jouf University, Sakaka, Al Jouf 72341, Saudi Arabia; ^8^Department of Pharmaceutics and Industrial Pharmacy, College of Pharmacy, Taif University, P.O. Box 11099, Taif 21944, Saudi Arabia

## Abstract

**Background:**

Different parts of *Taraxacum officinale* (L.) were used in traditional medicine in various parts of the world for the treatment of health problems, and they possess significant biological activities. The present study aimed to estimate phytochemical and biological activities of *T. officinale* using different extraction solvents.

**Methods:**

Methanolic, acetone, and n-hexane extracts of selected species were prepared, and ten secondary metabolites were examined using standard protocols. The antioxidant activity was performed using three *in vitro* methods, namely, DPPH assay, total reducing power (TRP) assay, and total antioxidant capacity (TAC). Toxicological analysis was done using the brine shrimp cytotoxic assay and radish seed phytotoxic assay.

**Results:**

The *T. officinale* methanolic extract showed the highest phenolic (178.27 ± 17.17 mg/GAE/g) and flavonoid (18.50 ± 1.64 mg QE/g) contents. Similarly, the methanolic extract also revealed the highest DPPH activity (32.80 ± 9.66 IC_50_), reducing potential (0.53 ± 0.02 mg/g), and TAC (19.42 ± 0.97 mg/g) as compared to the acetone and n-hexane extracts. The Pearson correlation analysis confirmed a strong positive correlation (*r* > 0.9) between total phenolic content (TPC), total flavonoid content (TFC), and all antioxidant assays. Furthermore, a heat map displayed the methanolic extract (red color) as a valuable source of phytochemicals and antioxidant agents. Moreover, the *T. officinale* methanolic extract also showed the highest (7.12 ppm) cytotoxic potential whereas both methanolic and acetone extracts were revealed as moderate phytotoxic agents when compared with the standard.

**Conclusion:**

The *T. officinale* methanolic extract exhibited comparatively notable phytochemicals that are actively involved in antioxidant activities and possess toxicological properties. This upholds the folkloric use of *T. officinale* as a possible source to develop natural plant-based drugs. Further investigations to isolate bioactive compounds and elements and on their safety need to be conducted.

## 1. Introduction

Azad Jammu and Kashmir (AJK) lies in the western part of the Himalayas, having an area of 13,269 km^2^. Poonch is located in the northern mountainous region of AJK and is situated 1750–2500 m above the sea level between 33–36 north latitude and 73–75 east longitude [1]. Rawalakot is the capital city of the Poonch district and has a temperate climate exhibiting annually an average rainfall of 1600 mm, and the temperature ranges from 3 to 26°C [2]. Most of the area has sandy clay soil and retains a greater moisture content (95% humidity) which enables plant growth efficiently [3]. Hilly areas possess a spectacular diversity of plant species and are contemplated as a treasured groove of medicinal plants [4]. The Poonch valley exhibits diversity in terms of medicinal plants owing to the presence of unique climatic conditions. Almost 70 species of the 430 wild species of this region have been explored medicinally till now [3]. Medicinal plants contain numerous bioactive compounds that are involved in curing ailments [5]. Most of the diseases prevailing in these areas are cured by folklore medicine as proposed by various herbalists [2].

*Taraxacum* genus comprises wild medicinal plants with almost 2500 species reported worldwide [6]. Some of these species are invasive and grown worldwide such as *Taraxacum officinale* var. *erythrospermum*; however, few are scientifically investigated nowadays. In the beginning, these species were used owing to the popular knowledge and experience from our ancestors. The first evidence of its therapeutic use is in Arabic medicine during the 10^th^ century to cure diseases of the liver and the spleen. Later on, Fuchs in 1953 described its use to treat diarrhea, blister, gout, and spleen and liver diseases [7]. Since the 12^th^ century, many researchers have diverted their attention from traditional knowledge toward the scientific explanations related to the mode of action of *Taraxacum* against diseases. Exploring the chemical composition and the action mechanism of this species against diseases using multiple *in vitro* and *in vivo* assays could establish its potential as a commercial herb [8, 9].

Uncontrolled production of reactive oxygen species (ROS) in the body causes oxidative stress which ultimately leads to diseases such as diabetes, aging, and myocardial infarctions [10]. To counteract this condition, cells have multiplex enzymatic and nonenzymatic elements. The molecules of the nonenzymatic system have the ability to cause enzyme inhibition and chelation of trace elements that are involved in ROS production through other antioxidant defenses [11]. Besides these molecules, phenolic and flavonoid compounds play a vital role in scavenging ROS by neutralizing free radicals and thus act as natural antioxidants [12]. These compounds act as antioxidants as they are stable intermediates and possess the ability to donate hydrogen or electrons [13]. The antioxidant capacity of phenols present in plant extracts is effective at low concentrations and is involved in the prevention of cardiovascular and cancer diseases [14, 15].

Moreover, phytochemicals also play a key role in plant adaptation under various growth conditions [16]. Plant growth could be reduced or adversely affected due to different weeds as they compete with them for available resources such as minerals, water, and space. Farmers use synthetic herbicides to control the growth of weeds which are harmful to human beings and also cause water and soil pollution [17]. Therefore, scientists have developed a great interest in exploring natural herbicides so that they could be utilized to enhance crop yield [18]. Moreover, plants also contain bioactive compounds which are toxic to organisms such as shrimps, and thus, the brine shrimp cytotoxicity test is usually recommended to determine the cytotoxic potential in plants [19].

Despite the longstanding vegetation growing in the Poonch valley, only a handful of species have been investigated scientifically. *Taraxacum* spp. (family Asteraceae) is commonly known as dandelion and is considered as a weed in several crops [20] while its leaves are rich in fibers, minerals, vitamins, and other bioactive compounds [21, 22]. Dandelion is a nontoxic herb that is exploited due to its diuretic, anti-inflammatory, and digestive stimulant properties [23]. It is extensively used for the treatment of eye diseases, cancer, osteoarthritis, and anemia [24]. In North American aboriginal medicine, decoctions and infusions of this herb are used for menstrual cramps, heartburn, dyspepsia, chest pain, and jaundice and to heal broken bones, bruises, swellings, and fractures [7]. In traditional Chinese medicine, dandelion is combined with other herbs to treat hepatitis and to increase immunity for upper respiratory tract infections, bronchitis, or pneumonia. *T. officinale* is also used to treat malaria in Venezuela, for toothache in Kosova, and for hypertension in Ghana [8]. Glufraz et al. [25] revealed the CCl_4_-induced hepatotoxicity potential of this plant in rats, and Khan et al. [26] determined the acaricidal potential of *T. officinale* against cattle tick *Rhipicephalus microplus* infestations. Moreover, Kenny et al. [27] reported the antimicrobial potential of methanolic root extracts of *T. officinale* against *Staphylococcus aureus* and *Bacillus cereus*.

Altogether, most of the previous studies have been focused on the ethnopharmacological potential of *T. officinale*, while few studies have been performed to confirm its biological potential. Hence, the present study was designed to explore the chemical composition, antioxidant, cytotoxic, and phytotoxic potential of *T. officinale* (L.) collected from the Poonch valley, by using multiple extraction solvents, to verify the rationale behind the use of this plant as a cure for various diseases.

## 2. Materials and Methods

### 2.1. Plant Collection and Extract Preparation

Fresh plant samples were collected from Rawalakot located in the Poonch district of Azad Kashmir. The plant sample was identified by Prof. Dr. Mir Ajab Khan (taxonomist), and a voucher number (QAU-AA-157) was assigned from the Herbarium of Pakistan, Quaid-i-Azam University (Islamabad). The plant material was washed, shade-dried, and powdered using an electric grinder. The plant material (20 gm) was extracted with 200 mL, each of methanol (polar), acetone (slightly polar), and n-hexane (nonpolar) solvents for 48 hours. Subsequently, filtration was carried out using a Whatman filter paper (Schleicher & Schell Kent, England), and the whole process was repeated twice. Subsequently, the obtained filtrate was concentrated using a rotary evaporator (Scilogex Re100-Pro, Keyland Court, Bohemia, US) and crude extracts were stored at 4°C for experimental analysis.

### 2.2. Preliminary Phytochemical Tests

Qualitative tests were performed to detect the presence of various secondary metabolites using standard protocols, namely, alkaline detection assay for flavonoids, Mayer's test for alkaloids, Salkowski test for glycosides, gelatin test for tannins, foam test for saponins, ferric chloride test for phenols, Libermann's test for terpenoids and steroids, and sodium chloride (NaCl) was added to the extract to observe anthocyanins (bluish color) and coumarins (yellow color) [28, 29].

### 2.3. Determination of Total Phenolic Content (TPC) and Total Flavonoid Content (TFC)

For TPC, each extract (20 *μ*L) was added in the Folin–Ciocalteu reagent (90 *μ*L) and then incubated for 5 minutes. Subsequently, 6% sodium carbonate (90 *μ*L) was mixed and absorbance was measured at 630 nm. Gallic acid (standard) was used to obtain the calibration curve [30]. In the case of TFC, the aluminium chloride colorimetric method was followed and the calibration curve was prepared using different concentrations of quercetin, a flavonoid standard [31]. The plant extract (20 *μ*L) was dissolved in 1 M potassium acetate (10 *μ*L), 10% aluminium chloride (Al_2_Cl_3_) (10 *μ*L), and distilled water (160 *μ*L), followed by incubation (30 minutes), and then, absorbance (405 nm) was noted.

### 2.4. Antioxidant Assays

#### 2.4.1. DPPH Scavenging Assay

The procedure described by Tepe et al. [32] was followed to observe the DPPH scavenging activity of selected extracts. In brief, each extract (10 *μ*L) was added to 0.004% of DPPH solution (190 *μ*L), and the final volume was made up to 200 *μ*L. The reaction mixture was placed in the dark for 30 minutes, and then, absorbance was measured at 517 nm wavelength. Ascorbic acid was used as a standard, and the scavenging activity was calculated by using the following formula:

(1) $$\begin{array}{c} \%\text{ scavenging activity}=\frac{{\text{Abs}}_{\mathrm{control}}-{\text{Abs}}_{\mathrm{sample}}}{{\text{Abs}}_{\mathrm{control}}}\times 100. \end{array}$$

#### 2.4.2. Total Reducing Power (TRP) Assay

About 200 *μ*L of each plant extract was added in potassium ferricyanide and phosphate buffer (500 *μ*L each) and then incubated at 50°C for 20 minutes. Subsequently, 500 *μ*L of trichloroacetic acid (TCA) was mixed followed by centrifugation (Model 2-6E, Sigma Laborzentrifugen, D-37520 Osterode am Harz, Germany) at 3000 rpm for 10 minutes. The supernatant was added in 0.1% ferric chloride (100 *μ*L), and then, absorbance was measured at 630 nm. Ascorbic acid was used as a control, and results were expressed as *μ*g AAE/mg of the extract [33].

#### 2.4.3. Total Antioxidant Capacity (TAC) Assay

For the TAC assay, Prieto's method [34] was followed with some modifications. In brief, the reaction mixture was prepared by adding the plant extract (50 *μ*L) in 4 mM ammonium molybdate (0.25 g), 28 mM sodium phosphate (1.68 g), 1.63 mL of H_2_SO_4_, and 50 mL of distilled water, followed by incubation at 95°C for 90 minutes. Then, the samples were cooled at room temperature, and absorbance was measured at 630 nm.

### 2.5. Toxicological Analysis

#### 2.5.1. Brine Shrimp' Cytotoxicity Assay

For the cytotoxicity assessment, Sirajuddin et al.'s [35] method was used with some modifications. Three working solutions (50, 100, and 150 *μ*g/mL) of each extract were prepared in dimethyl sulfoxide (DMSO) to ascertain the cytotoxicity potential of the selected extracts. *Artemia salina* eggs (Ocean Star, USA) were released in a bipartitioned tray filled with artificial saline water (3.8% sea salt in 1000 mL of distilled water; pH 7) and placed for incubation at 32°C. The lamp was used as a light source for the hatching of shrimps. After 24 hours, 10 shrimps were added to each vial along with different concentrations of the plant extract (5 *μ*L) and left for incubation for the next 24 hours. Subsequently, the number of alive shrimps was counted with the help of the naked eye, and (%) mortality and lethal concentration 50% (LC_50_) values were evaluated.

#### 2.5.2. Radish Seed Phytotoxicity Assay

For the radish seed phytotoxicity assay, the Arzu and Camper method [36] was followed with slight modifications. In this assay, the plant powder (50 mg) was dissolved in 5 mL of methanol, acetone, and n-hexane to prepare the test solution and water was used as a positive control. The solution was poured on the sterilized filter papers on the Petri plates inside the laminar hood (Model SC2-41 Changi South, Singapore) to avoid any contamination. After evaporation, 5 mL of distilled water was added, and then, 20 sterilized (using mercuric chloride) seeds were placed on each plate at a uniform distance. Petri plates were wrapped with parafilm and then incubated at 25°C in dim light. In the end, the percentage of seed germination and root length inhibition was determined using the following formula:

(2) $$\begin{array}{c} \text{root length inhibition }\%=\frac{\text{root length in test sample}}{\text{root length in control}}\times 100. \end{array}$$

### 2.6. Statistical Analysis

All assays were performed in triplicate, and mean ± standard deviation was determined. Least significant difference (LSD) was measured using Statistix 8.1 by performing analysis of variance (ANOVA), and IC_50_ values were calculated using the GraphPad-Prism 5 software. Pearson correlation analysis was performed between phytochemicals and antioxidant assays using Microsoft Excel, whereas a heat map was prepared to observe variability among extracts in terms of antioxidant activities. Furthermore, LC_50_ values were determined to ascertain the toxicity potential of extracts by using Probit analysis software [37].

## 3. Results

### 3.1. Phytochemical Analysis

Preliminary phytochemical tests revealed that secondary metabolites were present more abundantly in their methanolic extracts as compared to the acetone and n-hexane extracts. Saponins and tannins were not detected in n-hexane extracts, while glycosides were absent in acetone extracts. However, all other compounds were present strongly to moderately in most of the selected extracts (Table 1).

**Figure 1:**
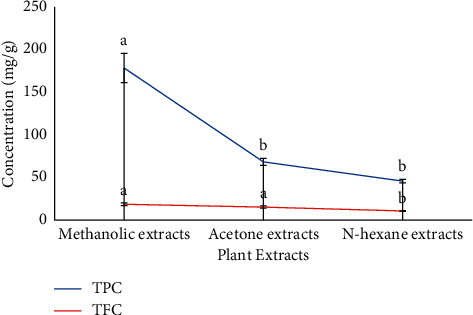
Total phenolic and flavonoid content detected in different extracts of *T. officinale*. The number represents mean ± SD (3n), and each letter (A and B) shows a significant difference at *p* < 0.05 as determined by LSD using Statistix 8.1.

Phenolic and flavonoid contents were determined quantitatively, and results showed that methanolic extracts exhibited the highest phenolic (178.273 ± 17.17 mg GAE/g) and flavonoid (18.5 ± 1.64 mg QE/g) contents as compared to the other extracts. Overall, phytochemicals were observed in decreasing order of methanolic extract > acetone extract > n-hexane extract, as shown in Figure 1.

### 3.2. Antioxidant Assays

**Figure 2:**
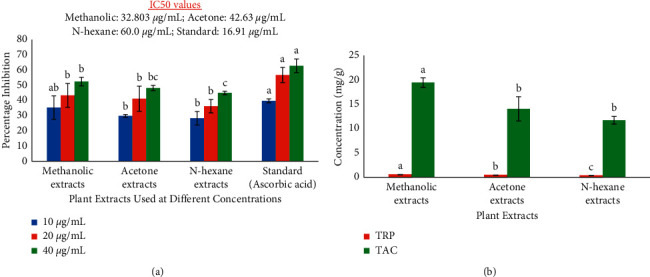
Antioxidant activity observed in *T. officinale* using different extraction solvents. The number represents mean ± SD (3n) and each letter (A–C) shows a significant difference at *p* < 0.05 as determined by LSD. (a) DPPH activity of the selected extracts measured at different concentrations. (b) Total reducing power (TRP) and total antioxidant capacity (TAC) of the selected plant extracts.

The antioxidant activity of *T. officinale* extracts was assessed using three methods, and among these, the DPPH activity was evaluated at three different concentrations to observe the percentage inhibition and IC_50_ values. Results revealed that all extracts exhibited a concentration-dependent percentage inhibition of free radicals in the DPPH assay. Among all samples, the highest activity was recorded in the methanolic extract (IC_50_ : 32.80 ± 9.66 *μ*g/mL), followed by acetone (IC_50_ : 42.63 ± 5.55 *μ*g/mL) and n-hexane (IC_50_ : 60.0 ± 8.37 *μ*g/mL) extracts, respectively. The highest antioxidant potential of methanolic and acetone extracts was also found to be statistically significant when compared with that of the standard ascorbic acid (Figure 2(a)).

Similarly, different extracts of *T. officinale* were further examined using the TRP and TAC assay. Higher absorbance indicated higher reducing potential of the plant extract (Figure 2(b)). Moreover, the total antioxidant capacity was also observed in descending order of methanolic extract (19.42 ± 0.97 mg/g) > acetone extract (14.01 ± 2.51 mg/g) > n-hexane extract (11.70 ± 0.79 mg/g) (Figure 2(c)).

### 3.3. Pearson Correlation Analysis and Heat Map Visualization

**Figure 3:**
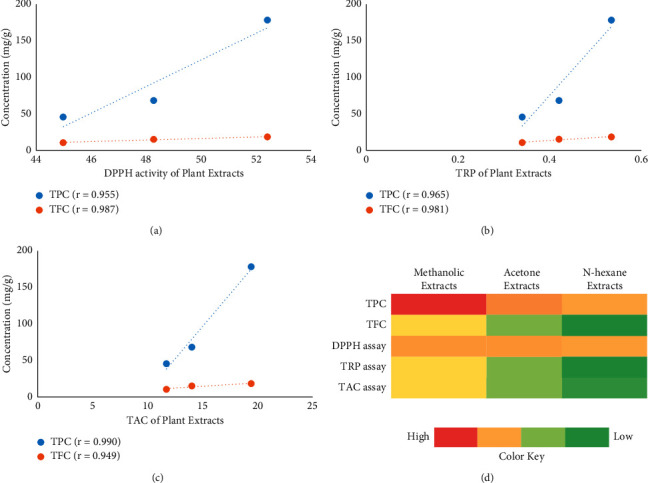
Pearson correlation analysis and the heat map of TPC, TFC, and three antioxidant assays as detected in different plant extracts. (a) Correlation between the DPPH assay and phytochemicals. (b) Correlation between TRP and phytochemicals. (c) Correlation between TAC and phytochemicals. (d) The heat map showing the comparison of antioxidant assays and phytochemicals among different extracts by displaying the highest (red) to lowest (green) activity in colors.

The correlation of antioxidant assays with phenolic and flavonoid compounds was observed which showed a strong positive correlation (*r* > 0.9) in all three antioxidant assays (Figures 3(a)–3(c)). Furthermore, a heat map was prepared to perceive differences between different kinds of extracts based on phytochemical tests and antioxidant assays. A higher activity was displayed vividly in red color, while green color indicated the lowest activity. The obtained data revealed that the extract prepared in a polar solvent (i.e., methanol) possessed the highest TPC and TFC along with antioxidant potential as examined by different methods, followed by other extracts made in slightly polar (i.e., acetone) and nonpolar (i.e., n-hexane) solvents, respectively (Figure 3(d)).

### 3.4. Toxicological Assays

To determine the cytotoxic potential of the selected extracts, brine shrimps were tested using different concentrations of extracts and vincristine sulphate was used as a standard. Among all samples, the extract prepared in methanol showed a potent cytotoxic effect exhibiting 7.122 ppm LC_50_ value followed by the extract made in acetone (i.e., 10.32 ppm LC_50_ value). However, the n-hexane extract revealed the lowest cytotoxic potential as it showed 14.02 ppm LC_50_ value (Table 2).

**Figure 4:**
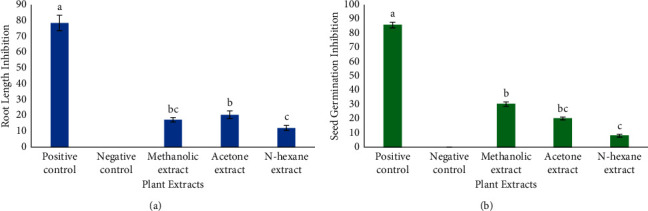
Phytotoxic potential of different extracts of *T. officinale*. The number represents mean ± SD (3n), and each letter (A–C) shows a significant difference at *p* < 0.05 as determined by LSD. (a) Root length inhibition. (b) Seed germination inhibition.

In the phytotoxic assay, paraquat was used as a standard while methanol was taken as a negative control. Regarding root length inhibition, the acetone extract showed the highest inhibition (20.465 ± 1.54%), whereas in the case of seed germination, methanolic extract was found to be most potent showing 30% inhibition in the seed germination. In general, all extracts revealed a moderate phytotoxic potential when compared with the standard and presented significant statistical differences as shown in Figures 4(a) and 4(b).

## 4. Discussion

For ages, nature has proved and served as a sumptuous repository of medicinal plants owing to the existing bioactive constituents that contribute toward the isolation of natural drugs [38]. Till now, numerous drugs have been explored from natural resources including medicinal plants via exploiting multiple techniques and approaches. These herbal medicines are effectively used against various diseases, especially in rural areas of less developed countries due to their presumed safety compared to conventional medicine [39, 40]. Keeping in view the importance of natural flora, there is a growing interest in exploring novel species so that they could be utilized at the industrial level. Hence, this present study aimed at investigating the chemical composition along with relative bioefficacy and toxicity potential of *T. officinale* collected from the Poonch valley (AJK).

Initially, *T. officinale* extracts were prepared using three solvents, namely, methanol (highly polar), acetone (slightly polar), and n-hexane (nonpolar) that were chosen by the difference in their polarity. Then, preliminary tests were conducted to ascertain the presence of ten secondary metabolites, and the results revealed that most of the tested compounds were abundantly present in the methanolic extract as compared to the acetone and n-hexane extracts of the selected plant. The presence of certain compounds in plant extracts can be attributed to the plant's physiological and biosynthetic reactions and the ecological conditions of the study area [41]. Moreover, the absence of few compounds in the acetone and n-hexane extract of *T. officinale* could be due to the low polarity of the solvent which impeded the extraction [42, 43]. Hence, it can be inferred that the variations were detected mainly due to the incompatible polarity indices of the solvents.

Among these bioactive compounds, phenolics and flavonoids are the dominant groups of phytochemicals that act as primary antioxidants or radical scavengers [44]. The concentration of these compounds varies in plants growing at different geographical locations [45]. In the current study, TPC and TFC were observed quantitatively in the selected extracts which displayed the highest concentration in the polar extract, i.e., 178.27 ± 17.17 mg/GAE/g and 18.50 ± 1.64 mg QE/g (Figure 1). Previously, Khan et al. [46] documented phenolic content ranging from 41.47 mg GAE/g to 691.6 mg GAE/g in *T. officinale* extracts which is comparable with our study which showed TPC ranging from 45.675 mg GAE/g to 178.27 mg GAE/g in the selected extracts. However, Kenny et al. [47] observed 228.72 mg GAE/g phenolic content in the ethyl acetate root extracts of *T. officinale* which is in disagreement with our study in which the whole plant was examined using different solvents.

Antioxidant potential of the selected extracts was compared as antioxidant activity aids in scavenging free radicals in a specific reaction medium. DPPH assay can be viewed as a milestone for the determination of the antioxidant activity of test samples [38]. Similarly, the reducing power and total antioxidant capacity assays are used to quantitatively assess the antioxidant efficacy of multiple extracts [19]. The results revealed that all extracts possess significant antioxidant potential, and this was observed in descending order of methanolic extract > acetone extract > n-hexane extract (Figure 2).

Our study correlates with the previous findings of Chon et al. [48] who documented a significant antioxidant potential in the methanolic extract of *T. officinale* collected from Korea. Similarly, our results are also coherent with the previous findings of Hu and Kitts [49] and Miłek et al. [50] who reported antioxidant activity in the water and ethyl acetate extracts of *T. officinale* leaves and flowers collected from Canada and Poland. Moreover, Kenny et al. [47] determined a DPPH activity of 227.72 ± 11.84 mg/g and a reducing potential of 463.06 ± 3.94 mg/g in the *T. officinale* root extract prepared in ethyl acetate. Thus, it can be proposed that *T. officinale* is equally effective against oxidative stress-related diseases regardless of geographical location and seasonal variations. These antioxidant assays provide a basis and rationale for using these extracts as antioxidant ingredients in various food and medicinal products. The higher phenolic content in the polar extract is also responsible for the higher antioxidant capacity of the selected plant extracts [19]. However, the decrease in antioxidant activity of slightly polar extracts can be due to the solubilization effect of polyphenols and their limited accessibility to DPPH radicals and other oxidized ions [49].

The obtained results revealed a good correlation between phytochemicals and antioxidant assays of the tested extracts (Figure 3) which suggests that phenolic and flavonoid compounds mainly affect the antiradical properties of the extracts. Herein, data indicated a strong positive (*r* > 0.9) correlation of phytochemicals with all antioxidant assays which is in parallel with the previous studies of Miłek et al. [50] who reported an *r* value above 0.8 between the antioxidant activities and TPC of *T. officinale* extracts. Therefore, it can be suggested that *T. officinale* collected from the Poonch valley also possesses a valuable reservoir of active compounds of pharmacological significance. In addition, differences between the three different kinds of *T. officinale* extracts were perceived by using a heat map which displayed high (red color) to low (green color) activities vividly (Figure 3(d)). Previously, Elhadef et al. [51] and Fernández-Poyatos et al. [52] also used a heat map to discriminate between different species and to observe the contribution of each extract to the biological activities.

In the current study, the brine shrimp assay was adopted to appraise the cytotoxicity potential of the selected extracts. The chemical or drug that kills nauplii is contemplated as a cytotoxic agent, and lethal concentration (LC_50_) values are used to express their cytotoxic level, whereby low values represent high cytotoxicity. According to Meyer's criteria, plant extracts with LC_50_ > 1000 *μ*g/mL are nontoxic, whereas plants with LC_50_ < 1000 *μ*g/mL are toxic [53]. Likewise, as per Clarkson's cytotoxicity criteria, the extract can be classified as nontoxic when LC_50_ > 1000 *µ*g/mL, slightly toxic when LC_50_ 500 to 999 *μ*g/mL, moderately toxic when LC_50_ 99 to 499 *μ*g/mL, and highly toxic when LC_50_ 0 to 100 *μ*g/mL [54]. Consequently, based on Meyer's and Clarkson's criteria, all extracts of *T. officinale* were highly toxic as they had LC_50_ values less than 100 *μ*g/mL (Table 2). The plant extracts with LC_50_ values < 20 *μ*g/mL exhibit more chances of producing anticancer compounds [55]. Hence, it can also be suggested that *T. officinale* can be used as a cytotoxic agent in particular conditions. Yet further toxicological studies could be conducted to establish the toxicity and safety profile of the selected extracts.

Moreover, weeds are the most important factors responsible for the reduction in crop yield. To counteract unwanted weeds, synthetic chemicals are used which are more or less associated with pollution, carcinogenesis, and high cost and thus their use is restricted [4, 18]. Accordingly, the search for alternative natural herbicides which are safe and cost-effective is recommended. In our study, both methanolic and acetone extracts were revealed as the most potent phytotoxic agents as they showed the highest root length inhibition (17.36 ± 2.48% and 20.46 ± 1.44%) and seed germination inhibition (30 ± 1.5% and 20 ± 1.0%) (Figure 4). So far, this is the first study on the brine shrimp cytotoxicity and radish seed phytotoxicity potential of *T. officinale* collected from the Poonch valley. It can be concluded from the present study that *T. officinale* can serve as a significant source of natural herbicides for weed control sustainably to enhance per acre yield, which warrants detailed investigations.

## 5. Conclusion

From this study, we came across a judgement that biological characteristics of plants are greatly influenced by the solvents used for extraction. As the solvent polarity reduces, the extraction process is hindered which eventually alters the chemical reactions inside the plants. The methanolic and acetone extract of *T. officinale* showed promising bioactive compounds and antioxidant activities which support its traditional use in industries. In contrast, the n-hexane (nonpolar) extract of *T. officinale* exhibits less antioxidant potential and is moderately toxic. Thus, polar extracts of the selected species can effectively serve as a natural source to formulate antioxidant and toxicological agents; however, these results cannot be applied directly to humans.

## 6. Recommendations

This research explored that the extracts of *T. officinale* prepared in polar solvents have higher medicinal value than the extracts prepared in other nonpolar solvents; however, further empirical investigations using *in vivo* models are needed. Besides this, isolation of pure compounds and their characterization are required to analyze their mode of action against various diseases. All-inclusive, the selected plant exhibits multiple properties and thus could be utilized by humans and animals in their dietary items and in preparing pharmaceutical products.

## Figures and Tables

**Table 1:** Qualitative phytochemical analysis of different extracts of *T. officinale*.

Phytochemicals	Methanolic extract	Acetone extract	n-Hexane extract
Alkaloids	++	+	+
Flavonoids	+++	++	++
Phenols	+++	++	++
Terpenoids	++	++	+
Steroids	++	+	+
Saponins	++	+	−
Tannins	+++	+	−
Anthocyanins	+++	++	+
Coumarins	+++	++	+
Glycosides	++	−	+

*Note.* +++: abundantly present; ++: moderately present; +: weakly present; −: absent.

**Table 2:** Percentage mortality and LC_50_ values observed in different extracts using the brine shrimp cytotoxic assay.

Plant extracts	Mortality (%) in probits at different doses					Slope	Intercept	R square	LC_50_	95% CI
	6	12	25	50	100					
Methanolic extract	5.00	5.25	5.52	6.28	—	1.334	3.863	0.917	7.122	3.642–13.926
Acetone extract	4.75	5.00	5.52	5.84	—	1.238	3.745	0.986	10.32	5.243–20.336
n-Hexane extract	4.48	5.00	5.25	5.84	5.84	1.167	3.660	0.942	14.12	7.066–28.238
Vincristine sulphate	5.00	5.52	5.52	5.84	—	0.816	4.462	0.867	4.608	1.677–12.662

LC_50_ : lethal concentration 50%; CI : confidence interval.

## Data Availability

The data used to support the findings of this study are included within the article. Any additional data will be delivered by the authors upon reasonable request.
